# Catheter-based Closure of a Post-infective Aortic Paravalvular Pseudoaneurysm Fistula With Severe Regurgitation After Two Valve Replacement Surgeries: A Case Report

**DOI:** 10.3389/fcvm.2021.693732

**Published:** 2021-08-20

**Authors:** Eustaquio Maria Onorato, Matteo Vercellino, Giovanni Masoero, Giovanni Monizzi, Federico Sanchez, Manuela Muratori, Antonio L. Bartorelli

**Affiliations:** ^1^Centro Cardiologico Monzino, IRCCS, University of Milan, Milan, Italy; ^2^Cardiovascular Disease Unit, IRCCS Ospedale Policlinico San Martino, Genova, Italy; ^3^Department Cardiology, Azienda Sanitaria Locale 1 Imperiese, Sanremo, Italy; ^4^Department of Biomedical and Clinical Sciences, “Luigi Sacco”, University of Milan, Milan, Italy

**Keywords:** prosthetic valve infective endocarditis, aortic valve surgery, paravalvular pseudoaneurysm, paravalvular leakage, catheter-based closure

## Abstract

**Backgsround:** Infective endocarditis (IE) of prosthetic valves is a dire complication of cardiac valve replacement surgery and is associated with high rates of morbidity and mortality.

**Case Summary:** A 72-year-old woman with multiple comorbidities underwent surgical replacement of the aortic valve with a mechanical prosthetic valve after recurrent IE. After nine months, IE recurred and the mechanical valve was surgically replaced with a bioprosthetic valve. Three years later, severe heart failure developed due to severe paravalvular leak (PVL) caused by an aortic annulus abscess complicated by a paravalvular pseudoaneurysm fistula (PPF). The patient was deemed at prohibitive surgical risk and a catheter-based PVL closure procedure was planned. However, the interventional procedure was delayed several months due to the Covid-19 pandemic with progressive heart failure worsening. Despite an acute satisfactory result of the PPF transcatheter closure and a significant clinical improvement, the patient died 10 months later due to multiorgan failure. It is likely that this was due, at least in part, to the long treatment delay caused by the unprecedented strain on the healthcare system.

**Discussion:** In patients at high surgical risk, early diagnosis and prompt interventional treatment of severe PVL are crucial for improving expectancy and quality of life. However, the recent outbreak of COVID-19 caused deferral of elective and semi-elective structural heart disease procedures (SHD) as in our case. Thus, a proactive and vigilant stance on managing SHD should be a priority even in the context of the COVID-19 pandemic.

## Introduction

Infective endocarditis (IE) of prosthetic valve endocarditis is a life-threatening complication of cardiac valve replacement surgery that is associated with significant morbidity and mortality (1, 2). There is a high risk of IE in patients with bioprosthetic compared to mechanical valves, both within and beyond 1 year after surgery (3).

We report a case of a paravalvular pseudoaneurysm fistula (PPF) with severe paravalvular leak (PVL) after IE of a bioprosthetic aortic valve causing severe heart failure in a 72-year-old woman with multiple comorbidities. The patient, who was at prohibitive surgical risk, underwent successful PPF transcatheter closure. However, the catheter-based treatment was delayed due to the COVID-19 pandemic, and the clinical condition progressively worsened after the procedure leading to patient death of multiorgan failure.

## Case Presentation

A 72-year-old obese (BMI 33 Kg/m^2^) woman underwent aortic valve replacement with a mechanical valve prosthesis (Sorin Slimline 23 mm) after developing IE complicated by a left coronary cusp abscess leading to severe aortic regurgitation and heart failure. History included hypertension, dyslipidemia, type II diabetes, chronic obstructive pulmonary disease, and two-vessel coronary artery disease that was treated with drug-eluting stent implantation. Ten months after surgery, the patient was hospitalized because of heart failure with complex ventricular arrhythmias. Prosthetic valve dehiscence due to IE with severe aortic regurgitation was diagnosed. She received an implantable cardioverter defibrillator and underwent surgical removal of the aortic mechanical prosthesis, extensive debridement of the infected paravalvular tissue and implantation of a bioprosthetic aortic valve (Sorin Crown PRT 23 mm). The post-operative course was complicated by recurrent cardiac tamponade requiring surgical revision and sepsis due to staphylococcus hemolyticus requiring prolonged hospitalization and rehabilitation.

**Figure 1:**
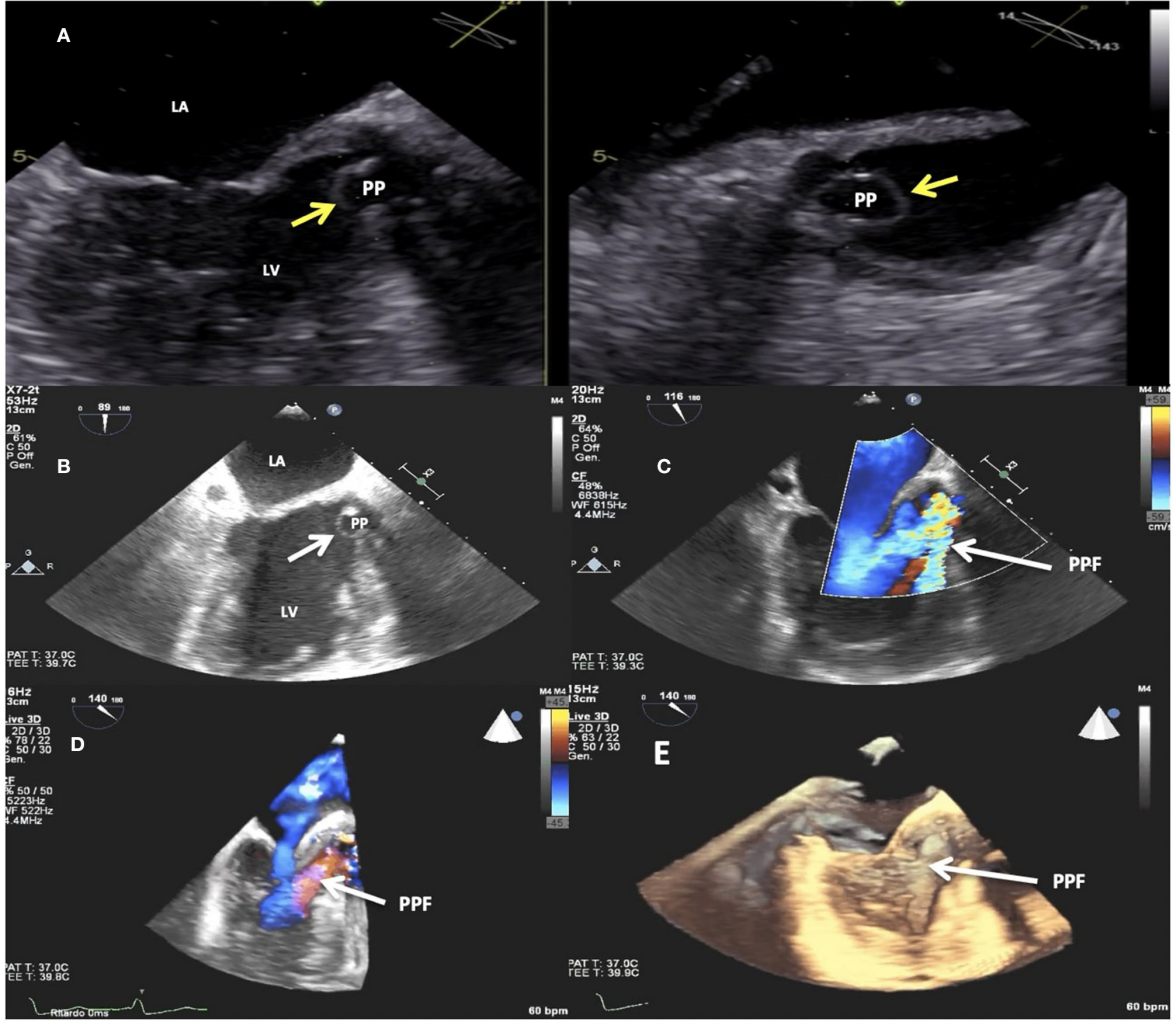
Transesophageal echocardiogram (TEE). 2D imaging showing the paravalvular pseudoaneurysm (yellow arrows) protruding in diastole into the left ventricle outflow tract **(A,B)**. 2D **(C)** and 3D **(D,E)** TEE color Doppler showing the paravalvular pseudoaneurysm (white arrows) protruding in diastole into the left ventricle outflow tract and the paravalvular pseudoaneurysm fistula causing severe regurgitation. LV, left ventricle; LA, left atrium; PP, paravalvular pseudoaneurysm; PPF, paravalvular pseudoaneurysm fistula.

**Figure 2:**
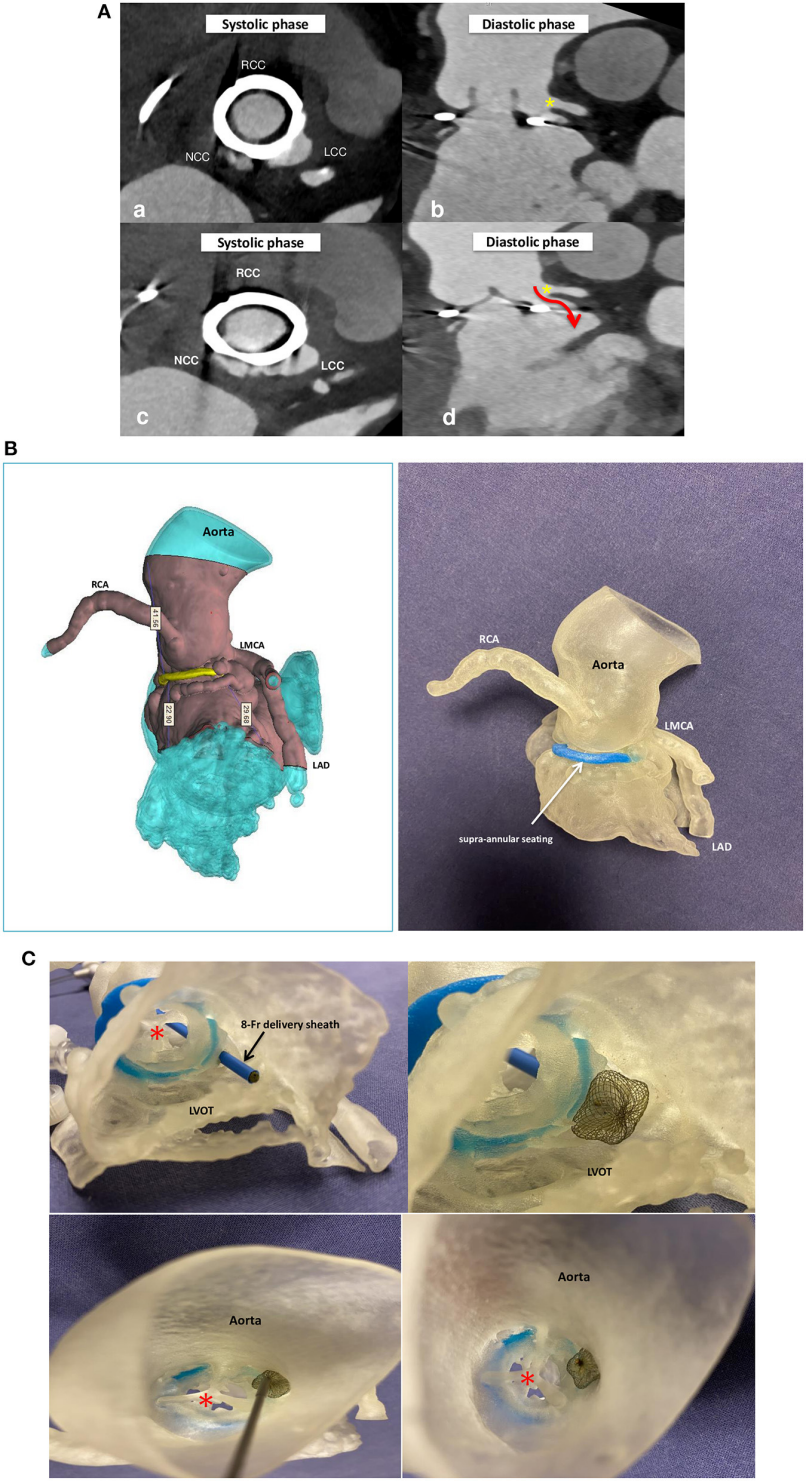
Multidetector computed tomography angiography (MDCTA) scans **(A)** with acquired frames in systolic (a,c) and diastolic phase (b,d) confirming the presence of the paravalvular pseudoaneurysm fistula located at the left coronary cusp in close proximity of the left main coronary artery (yellow star) and protruding in diastole into the left ventricle outflow tract (red arrow). Silicone printed 3D heart model made on the basis of MDCTA scan reconstruction **(B)**. Silicone 3D model seen from the left ventricle outflow tract (upper panels) and from the ascending aorta (bottom panels) and used to mimic the implantation of a self-expanding double-disc 5-mm square twist Occlutech PLD, with a distal left ventricle disc of 10 mm and a proximal aortic disc of 8.5 mm. Note that the device does not impinge on the bioprosthetic aortic valve (red star). RCC, right coronary cusp; LCC, left coronary cusp; NCC, non-coronary cusp; RCA, right coronary artery; LMCA, left main coronary artery; LAD, left anterior descending; LVOT, left ventricle outflow tract.

**Figure 3:**
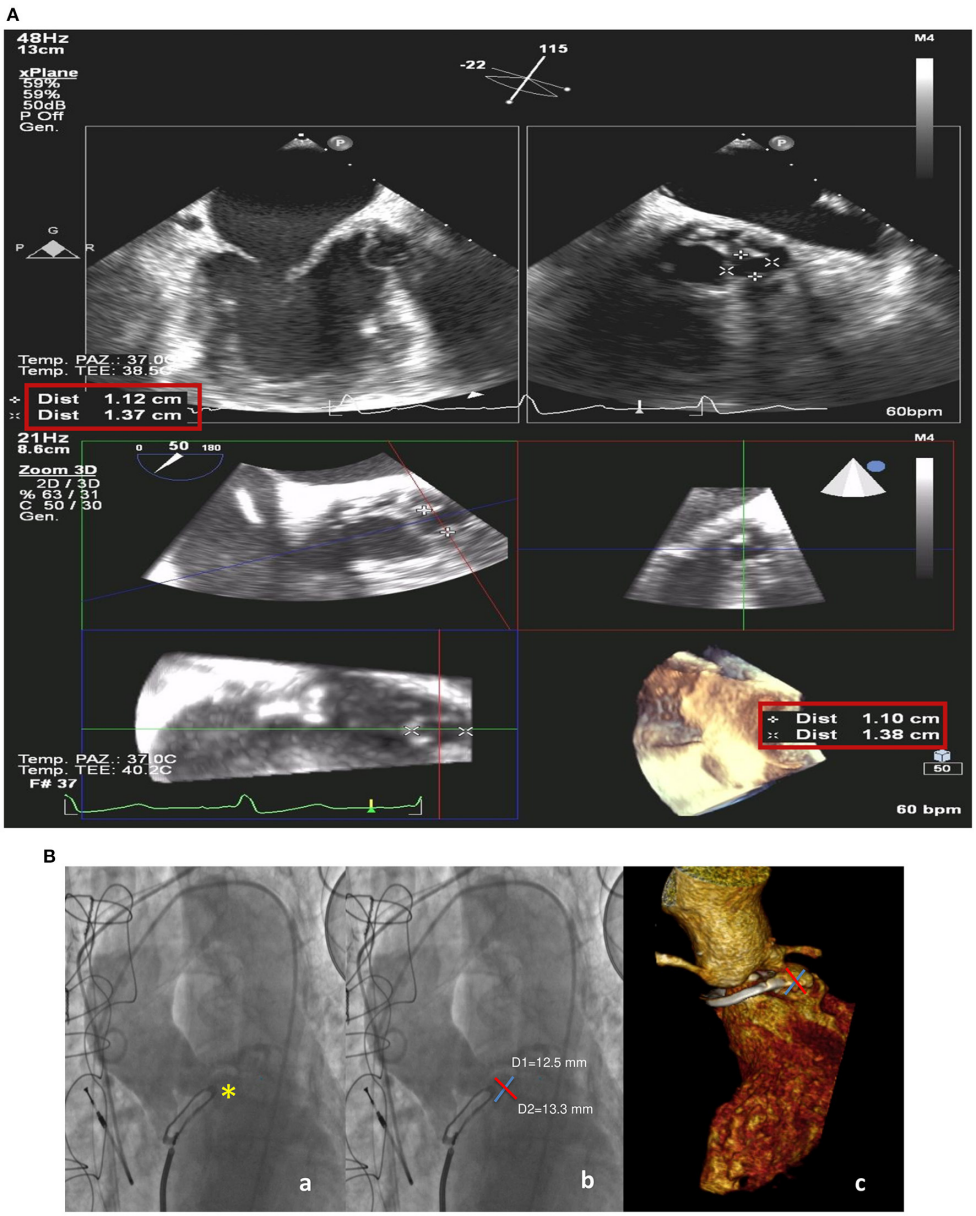
Intraprocedural transesophageal echocardiogram **(A)**. 2D X-plane imaging (upper panels) and 3D (bottom line) with measurements of the paravalvular pseudoaneurysm fistula. Preprocedural ascending aorta angiogram and volume-rendering three-dimensional (3D) multidetector computed tomography angiography (MDCT) **(B)** showing the paravalvular pseudoaneurysm (yellow star) (a) and its measurements on the two orthogonal axes from angiographic (b) and MDCT images (c).

**Figure 4:**
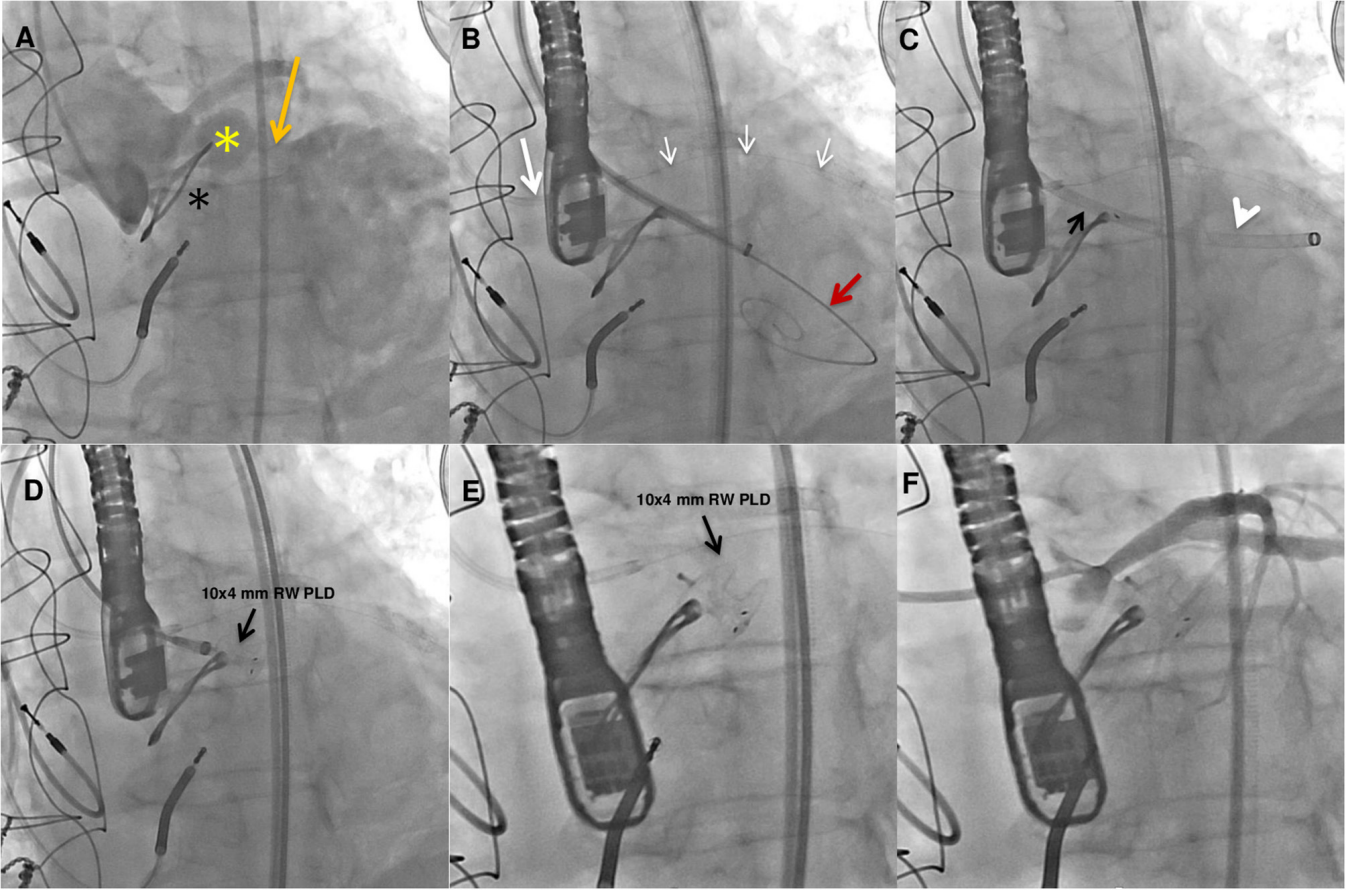
Fluoroangiographic procedural steps. Baseline ascending aorta angiogram showing the paravalvular pseudoaneurysm (yellow star) adjacent to the bioprosthetic aortic valve (black star) and the fistula (orange arrow) causing severe regurgitation in the left ventricle **(A)**. A preshaped extra small guidewire (Safari^2^) was placed across the pseudoanuerysm fistula in the left ventricle and an 8-Fr delivery sheath was advanced over it. Protection of the left main coronary artery was obtained with a coronary guidewire (small white arrows) advanced in the left coronary artery through an extra back up guiding catheter (white arrow) **(B)**. The PLD (black arrow) was advanced into the 8-Fr delivery sheath (white arrowhead) **(C)**. A 10 × 4-mm rectangular waist (RW) PLD (black arrow) still anchored and finally deployed in the correct position **(D,E)**. Postprocedure angiography showing mild to moderate regurgitant jet and no impingement on the left main coronary artery **(F)**.

Three years later, she was hospitalized because of congestive heart failure. Two-dimensional and three-dimensional (2D/3D) transesophageal echocardiography (TEE) showed a well-functioning bioprosthetic valve and a PPF protruding in diastole into the left ventricle outflow tract with severe PVL (Figures 1A–E). Diastolic reversal flow with high velocity (>20 cm/s) in the descending aorta confirmed regurgitation severity. The PPF size was about 9 × 7 mm, with a neck size of 5 mm and an EROA of 0.5 cm^2^. Multidetector computed tomography (MDCT) (Figure 2A) confirmed the PPF location under the left coronary cusp close to the left main coronary artery ostium. A silicone-printed 3D heart model was made based on MDCT reconstruction (Figure 2B, Supplementary Figure 1)to better understand PPF anatomy and its relationship with the surrounding structures and for planning “ex vivo” transcatheter implantation of the most appropriate occluding device (Figure 2C). This turned out to be a self-expanding double-disc 5-mm square twist PLD (PLD, Occlutech, Helsingborg, Sweden) with a 10-mm left ventricular disc and an 8.5-mm aortic disc. The patient was discharged to be referred to our tertiary cardiovascular center for transcatheter PPF closure. However, due to the impact of the COVID-19 pandemic on the national health system, elective interventional procedures were restrained in all Italian centers and the patient hospitalization was put on hold (4). During the waiting time, she was hospitalized again for congestive heart failure complicated by ventricular fibrillation, properly treated by ICD. The hemodynamic condition was unstable and required intravenous diuretics, inotropes and non-invasive ventilation. Ecocardiography documented a left ventricle ejection fraction of 40% with a moderate to severe mitral regurgitation. Two months later, the patient was finally transferred to our tertiary hub cardiovascular center. She was symptomatic for dyspnea at rest and was treated with high-dose of i.v. furosemide. Three days after admission, hyperpyrexia (up to 38.4°C) developed and blood culture showed the presence of staphylococcus aureus. She was treated with piperacillin/tazobactam and vancomycin with clinical benefit. At the same time, severe anemia occurred likely due to hemolysis through the PVL requiring blood transfusion. After 15 days of diuretic and antibiotic therapy, the patient was relatively stable with clinical improvement and furosemide dosage was reduced. However, a new episode of acute heart failure occurred requiring high-dose diuretics, dobutamine and non-invasive ventilation that rapidly restored an acceptable hemodynamic state. The decision was made by our multidisciplinary heart team to offer the only possible therapeutic option, a percutaneous PVL closure, due to the prohibitive surgical risk of the patient. Written informed consent, after detailed explanation, was obtained from the patient. The procedure was carried out in a hybrid operating room under general anesthesia, continuous real time 2D/3D TEE and fluoroscopic guidance. Ascending aorta angiogram showed the PPF adjacent to the bioprosthetic aortic valve with severe PVL (Supplementary Video 1). The PPF was measured with TEE and angiography that showed a diameter of 11 × 13.8 mm (Figure 3A) and 12.5 × 13.3 mm (Figure 3B), respectively. A pre-shaped extra small exchange guidewire (Safari^2^) was positioned through the PPF into the left ventricle apex and an 8-Fr delivery sheath was advanced over the wire. The left main coronary artery was protected with a 0.014” guidewire advanced through a 3.5 extra back up guiding catheter (Figures 4A–C). Based on the measurements made with TEE and angiography, a bigger PDL (10 × 4 mm), different both in shape (rectangular) and in type of connection (waist), with a 19-mm distal LV disc and 17-mm proximal aortic disc was advanced into the LV (Figure 4D; Supplementary Figure 2) and deployed under continuous fluoroscopic, angiographic and 2D/3D TEE guidance. Correct device alignment was achieved thanks to the rotational feature of the Occlutech PLD with significant decrease of the regurgitant jet and without impingement of the device on the prosthetic aortic leaflets. A pull-and-push maneuver was performed to check PLD stability and the device was successfully deployed (Figure 4E). Final TEE and angiographic assessment confirmed the PLD stable position with moderate residual leak (Figure 4F; Supplementary Videos 2–4). After the procedure, the patient remained hemodynamically stable, antibiotic therapy was continued for 15 days and she was then transferred to a rehabilitation center with the indication to maintain long-term oral antibiotics. However, 1 month later the clinical condition worsened again with congestive heart failure and deterioration of renal function requiring hospitalization. On admission, 2D TEE confirmed a satisfactory result of the PPF percutaneous closure with mild-to-moderate residual leak and mitral regurgitation graded as moderate to severe. Irreversible clinical worsening occurred and the patient died of multiorgan failure 2 days later.

## Discussion

Infection of a prosthetic cardiac valve, the incidence of which is about 2.5% among patients having undergone valve replacement, is a serious complication with considerable morbidity and mortality (5–7). Paravalvular leak (PVL) is another potential complication of both mechanical and bioprosthetic valve implants, with an incidence of 2–10% in the aortic position and 7–17% in the mitral position, with varying degrees of severity (8). It may cause heart failure, hemolysis or both and has been shown to be an independent risk factor for poorer long-term outcome (9). Significant PVL is also a marker of poor long-term outcome after transcatheter aortic valve replacement for both balloon-expandable and self or mechanical-expanding valves (10). Over time, percutaneous treatment has emerged as a reliable and cost-effective alternative to surgery (11). Pseudoaneurysm formation due to IE may cause a perivalvular abscess cavity that over time may perforate into the LV creating significant PVL (12, 13). Pseudoaneurysms have been increasingly recognized with echocardiography. Use of 2D/3D TEE color Doppler has high diagnostic sensitivity in detecting the precise location and size of pseudoaneurysms and their relationship with the surrounding structures. Complementary imaging modalities like MDCT and cardiac magnetic resonance are also used for better morphologic and functional assessment (14). Additionally, our case illustrates the potential of 3D cardiac modeling for a thorough evaluation of the anatomical structure of these defects and their relationship to adjacent structures (15, 16). Indeed, a critical aspect of these procedures is the choice of the most appropriate device in terms of size and morphology that best fits the complex anatomy of a PPF, like the one that developed in our patient, without interfering with the prosthetic valve function or impinging on coronary ostia. In our case, 3D printed modeling allowed to test the device before the interventional procedure limiting residual leak and preventing serious complications. However, 3D printing is an expensive and demanding technology and its benefit and cost-effectiveness should be investigated in future trials.

Finally, our case demonstrates that delay in treating patients in need of structural heart disease (SHD) intervention is associated with unavoidable morbidity and mortality because many of them have life-threatening conditions. The recent outbreak of COVID-19 is placing an unprecedented strain on patients, physicians and world healthcare systems that resulted in deferral of elective and semi-elective SHD procedures. It is likely that delay in our patient treatment had a potential role in progressive worsening of hemodynamic and clinical conditions, infection recurrence and irreversible multiorgan failure leading to death despite a successful percutaneous treatment of the PPF.

## Conclusion

This case indicates that catheter-based closure of PPF may be a feasible and safe alternative to surgical repair, particularly in patients with prohibitive risk for redo surgery. In complex cases, 3D cardiac modeling may help in better planning and guiding the interventional procedure. Patients with heart failure due to PVL should be treated as soon as possible to prevent multiorgan failure. Thus, a proactive and vigilant stance on managing SHD is crucial, especially in the context of the COVID-19 pandemic, when the risk of overlooking severely sick patients or postponing life-saving treatments is high.

## Data Availability Statement

The raw data supporting the conclusions of this article will be made available by the authors, without undue reservation.

## Ethics Statement

The studies involving human participants were reviewed and approved by Centro Cardiologico Monzino, IRCCS, University of Milan, Milan, Italy. The patients/participants provided their written informed consent to participate in this study. Written informed consent was obtained from the individual(s) for the publication of any potentially identifiable images or data included in this article.

## Author's Note

This paper was the original work of the authors who have all seen and approved of the paper and authorship. The article has not been published elsewhere and is not under consideration in any other journals.

## Author Contributions

All authors listed have made a substantial, direct and intellectual contribution to the work, and approved it for publication.

## Conflict of Interest

EO is a consultant for Occlutech, manufacturer of the device. The remaining authors declare that the research was conducted in the absence of any commercial or financial relationships that could be construed as a potential conflict of interest.

## Publisher's Note

All claims expressed in this article are solely those of the authors and do not necessarily represent those of their affiliated organizations, or those of the publisher, the editors and the reviewers. Any product that may be evaluated in this article, or claim that may be made by its manufacturer, is not guaranteed or endorsed by the publisher.

## Supplementary Material

Supplementary Figure 1 3D cardiac modeling for a thorough evaluation of the anatomical structures and their relationship to adjacent structures.

Supplementary Figure 2 Occlutech rectangular waist PLD specifications.

Supplementary Video 1 Baseline ascending aorta angiogram showing the paravalvular pseudoaneurysm fistula adjacent to the bioprosthetic aortic valve and close to the left main coronary artery with severe regurgitation in the left ventricle.

Supplementary Video 2 Final angiography confirming the stable position of the 10 × 4-mm rectangular waist PLD with mild-to-moderate residual regurgitation and no impingement on the left main coronary artery and bioprosthetic valve.

Supplementary Video 3 and 4 Final 3D TEE confirming the correct position of the 10 × 4-mm rectangular waist PLD with mild-to-moderate residual regurgitant jet. Note the proximal disc parallel to the supra-annular ring with no impingement on the bioprosthetic leaflets.
