# Corrigendum: Catheter-based Closure of a Post-infective Aortic Paravalvular Pseudoaneurysm Fistula With Severe Regurgitation After Two Valve Replacement Surgeries: A Case Report

**DOI:** 10.3389/fcvm.2021.788004

**Published:** 2021-10-26

**Authors:** Eustaquio Maria Onorato, Matteo Vercellino, Giovanni Masoero, Giovanni Monizzi, Federico Sanchez, Manuela Muratori, Antonio L. Bartorelli

**Affiliations:** ^1^Centro Cardiologico Monzino, IRCCS, University of Milan, Milan, Italy; ^2^Cardiovascular Disease Unit, IRCCS Ospedale Policlinico San Martino, Genova, Italy; ^3^Department Cardiology, Azienda Sanitaria Locale 1 Imperiese, Sanremo, Italy; ^4^Department of Biomedical and Clinical Sciences, “Luigi Sacco”, University of Milan, Milan, Italy

**Keywords:** prosthetic valve infective endocarditis, aortic valve surgery, paravalvular pseudoaneurysm, paravalvular leakage, catheter-based closure

In the original article, there was an error.

**After 10 years, IE recurred and the mechanical valve was surgically replaced with a bioprosthetic valve. Ten years later, severe heart failure developed due to severe paravalvular leak (PVL) caused by an aortic annulus abscess complicated by a paravalvular pseudoaneurysm fistula (PPF)**.

A correction has been made to ***ABSTRACT***, ***Case Summary, lines 3–4***:

**After nine months, IE recurred and the mechanical valve was surgically replaced with a bioprosthetic valve. Three years later, severe heart failure developed due to severe paravalvular leak (PVL) caused by an aortic annulus abscess complicated by a paravalvular pseudoaneurysm fistula (PPF)**.

The authors apologize for this error and state that this does not change the scientific conclusions of the article in any way. The original article has been updated.

## Publisher's Note

All claims expressed in this article are solely those of the authors and do not necessarily represent those of their affiliated organizations, or those of the publisher, the editors and the reviewers. Any product that may be evaluated in this article, or claim that may be made by its manufacturer, is not guaranteed or endorsed by the publisher.

