# Supplementary figures and images for: Catheter-based Closure of a Post-infective Aortic Paravalvular Pseudoaneurysm Fistula With Severe Regurgitation After Two Valve Replacement Surgeries: A Case Report
Source: Front Cardiovasc Med. 2021 Aug 20;8:693732. doi: 10.3389/fcvm.2021.693732 (PMC8419236; doi:10.3389/fcvm.2021.693732)

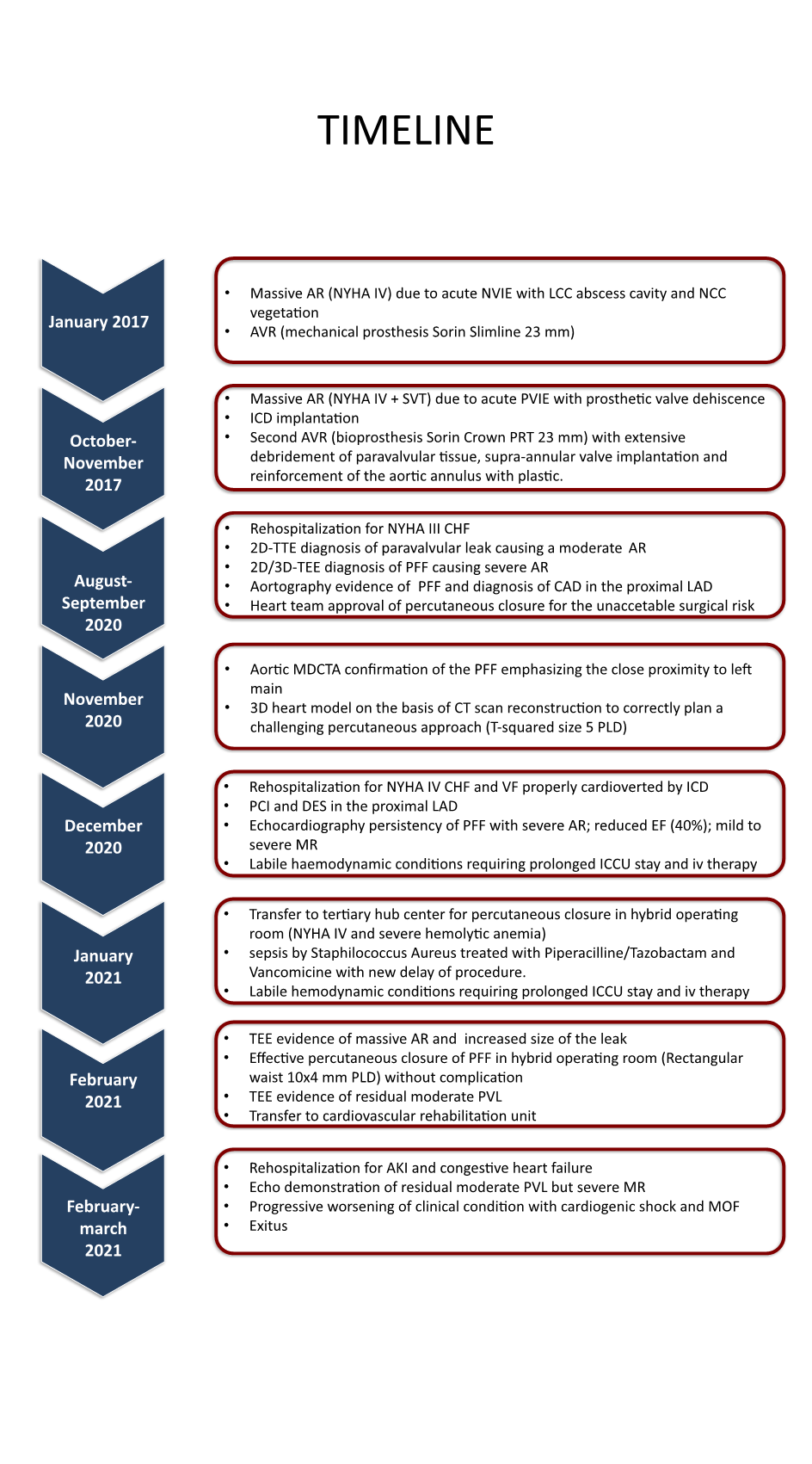

Supplement: Supplementary file 1 [file Image_3.TIFF]

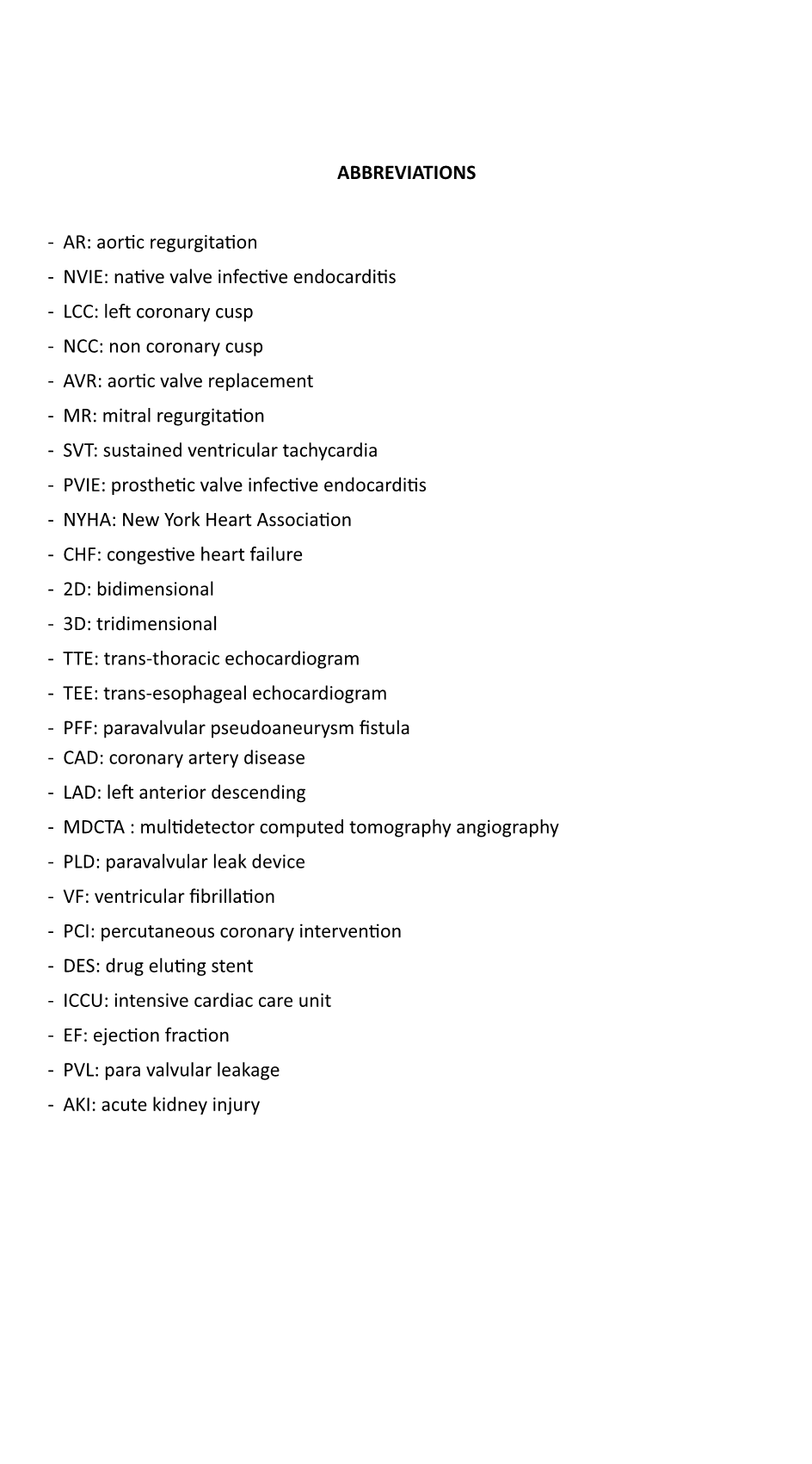

Supplement: Supplementary file 2 [file Image_4.TIFF]

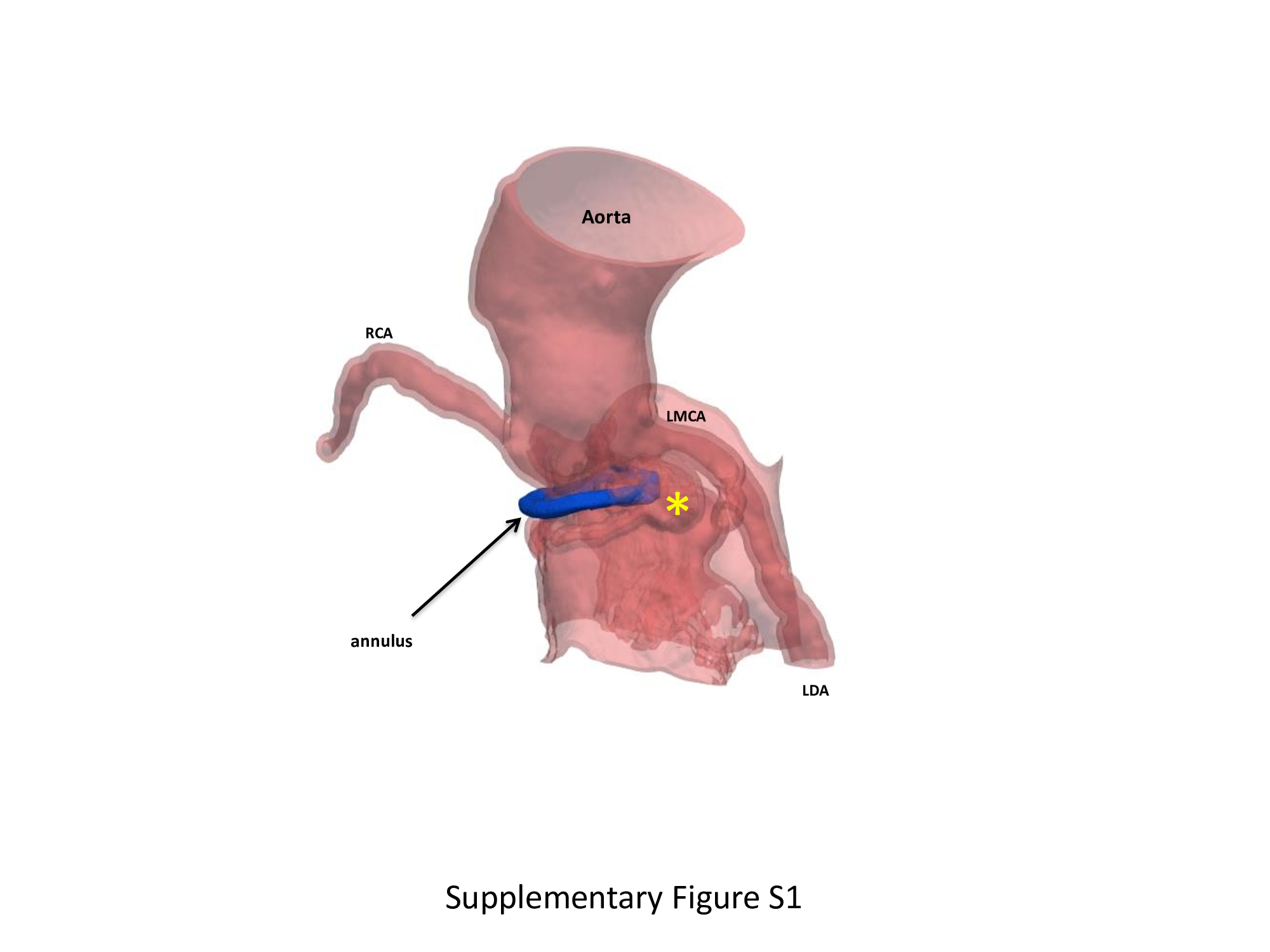

Supplement: Supplementary Figure 1 — 3D cardiac modeling for a thorough evaluation of the anatomical structures and their relationship to adjacent structures. [file Image_1.TIFF]

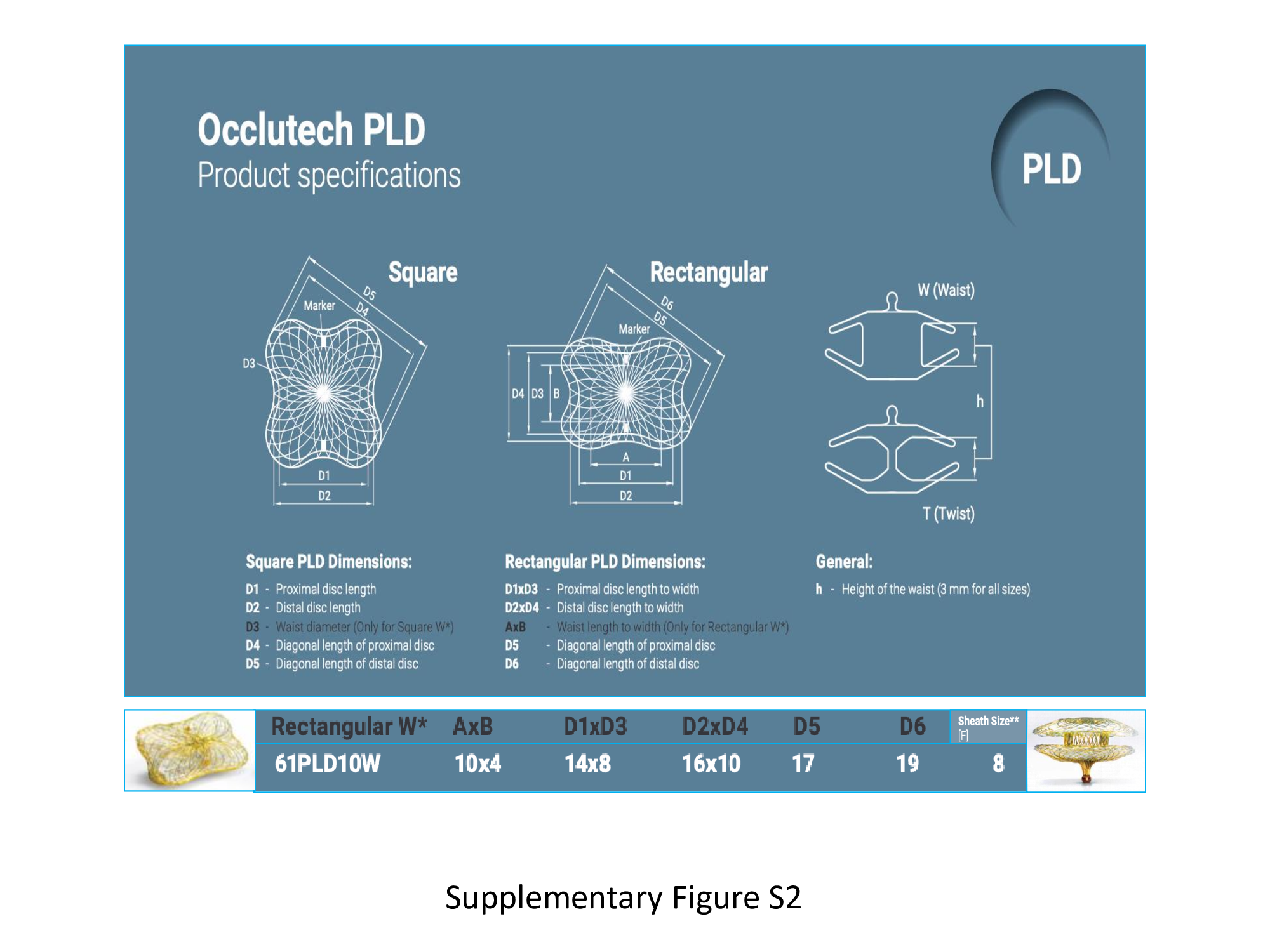

Supplement: Supplementary Figure 2 — Occlutech rectangular waist PLD specifications. [file Image_2.TIFF]
